# *P. falciparum* cpn20 Is a Bona Fide Co-Chaperonin That Can Replace GroES in *E. coli*

**DOI:** 10.1371/journal.pone.0053909

**Published:** 2013-01-10

**Authors:** Anna Vitlin Gruber, Shahar Nisemblat, Gal Zizelski, Avital Parnas, Ron Dzikowski, Abdussalam Azem, Celeste Weiss

**Affiliations:** 1 George E. Wise Faculty of Life Sciences, Department of Biochemistry and Molecular Biology, Tel Aviv University, Ramat Aviv, Israel; 2 Department of Microbiology and Molecular Genetics, The Institute for Medical Research Israel - Canada, The Kuvin Center for the Study of Infectious and Tropical Diseases, The Hebrew University-Hadassah Medical School, Jerusalem, Israel; University of California Davis, United States of America

## Abstract

Human malaria is among the most ubiquitous and destructive tropical, parasitic diseases in the world today. The causative agent, *Plasmodium falciparum*, contains an unusual, essential organelle known as the apicoplast. Inhibition of this degenerate chloroplast results in second generation death of the parasite and is the mechanism by which antibiotics function in treating malaria. In order to better understand the biochemistry of this organelle, we have cloned a putative, 20 kDa, co-chaperonin protein, Pf-cpn20, which localizes to the apicoplast. Although this protein is homologous to the cpn20 that is found in plant chloroplasts, its ability to function as a co-chaperonin was questioned in the past. In the present study, we carried out a structural analysis of Pf-cpn20 using circular dichroism and analytical ultracentrifugation and then used two different approaches to investigate the ability of this protein to function as a co-chaperonin. In the first approach, we purified recombinant Pf-cpn20 and tested its ability to act as a co-chaperonin for GroEL *in vitro*, while in the second, we examined the ability of Pf-cpn20 to complement an *E. coli* depletion of the essential bacterial co-chaperonin GroES. Our results demonstrate that Pf-cpn20 is fully functional as a co-chaperonin *in vitro*. Moreover, the parasitic co-chaperonin is able to replace GroES in *E. coli* at both normal and heat-shock temperatures. Thus, Pf-cpn20 functions as a co-chaperonin in chaperonin-mediated protein folding. The ability of the malarial protein to function in *E. coli* suggests that this simple system can be used as a tool for further analyses of Pf-cpn20 and perhaps other chaperone proteins from *P. falciparum*.

## Introduction

Malaria is an infectious disease caused by *Plasmodium falciparum* parasites that infects hundreds of millions of people worldwide, mostly in tropical and semitropical climates. It is estimated that on the order of 1 million deaths are caused each year by malarial infection, the majority of the victims being children in sub-Saharan Africa [1]. The importance of understanding *P. falciparum* biochemistry cannot be overestimated. Recent molecular evolution studies have shown that a number of alveolate species, including malaria, have evolved from photosynthetic red algae [2]. As such, these retain a vestigial, non-photosynthetic plastid known as the “apicoplast”. Although no longer photosynthetic, this organelle is predicted to import over 500 nuclear-encoded proteins, most of which contain a bipartite targeting sequence directing the proteins first to the lumen of the ER and from there to the apicoplast stroma [3], [4]. Based on sequence similarity, these proteins are thought to participate in biosynthesis of isoprenoid, fatty acid, heme and iron-sulfur clusters [5], [6]; although a recent study has indicated that only the isoprenoid synthesis function is critical for survival of blood-stage parasites [7]. Additional proteins of known housekeeping function were also identified in the apicoplast, such as DNA polymerase, gyrase, ribosomal proteins and molecular chaperones [8]. The latter include one gene for a 60 kDa chaperonin subunit and one gene for a co-chaperonin composed of 20 kDa subunits [9], [10].

Chaperonin proteins of 60 and 10 kDa subunits work together as a protein folding apparatus in the majority of living cells, in order to fold proteins following translation, translocation or stress-induced denaturation [11], [12]. The prototypical *E. coli* GroEL chaperonin is an extremely stable tetradecamer composed of 60 kDa subunits, which refolds client proteins with the help of its heptameric co-chaperonin, GroES, comprising 10 kDa subunits. Although a homologous system in chloroplasts was shown to exhibit a degree of structural and functional similarity to that of the bacterial system, a number of characteristics come to differentiate between the two [13], [14]. The most relevant difference for this study is the fact that plants contain two distinct structural types of GroES-like co-chaperonin proteins. The gene of one, cpn10, encodes for a 10 kDa subunit, which is known to form heptameric ring-shaped oligomers, while the second gene, cpn20, encodes for a “double–domained”cpn10 protein [15]–[17]. The latter gene consists of two linked homologous cpn10 sequences. It is still not completely clear how these subunits oligomerize at the protein level [18], however, it has been reported that the cpn20 subunits may assemble into tetrameric species *in vitro* [19], or form hetero-oligomers with cpn10 subunits [20]. Until recently, the cpn20 protein was known to exist only in chloroplasts of algae and plants. The discovery of a 20 kDa co-chaperonin in *P. falciparum* (Pf-cpn20) came about as the result of the malarial genome sequencing, and can be understood in light of the algal origin of the parasite [2], [10]. In systems containing only one cpn60 and one cpn10 (such as *E. coli,* or *S. cerevisiae*) these proteins are essential for survival of the organism [21], [22]. Since the apicoplast contains only one cpn20 and one cpn60 protein, it seems reasonable to assume that these are similarly vital to protein folding in this malarial organelle. However, co-chaperonin function of Pf-cpn20 has not yet been demonstrated and preliminary reports cast doubt on its ability to function in this capacity [10].

In this study, we cloned the Pf-cpn20 gene and overexpressed the protein in *E. coli*. The protein was purified and characterized *in vitro* by circular dichroism (CD), analytical ultracentrifugation (AU) and a malate dehydrogenase (MDH) refolding assay. Additionally, the function of the protein was characterized *in vivo* in *E. coli*. We provide the first evidence that Pf-cpn20 is fully functional at assisting protein folding in the place of GroES both *in vitro* with GroEL and *in vivo* in *E. coli*, thereby acting as a bona fide co-chaperonin.

## Materials and Methods

### Plasmids and Strains

Cpn20 from *P. falciparum* (PF3D7_1333000) was amplified by PCR, using cDNA synthesized from VBH-stage mRNA as a template. The forward and reverse primers were designed to include Nde1 and Xho1 restriction sites, respectively, for cloning into the pET22b+ plasmid.

Forward: PfCpn20F_2009∶5′-TTA CATATG TATAAAATTGATAATAAAGTAATAAGAGGTCCTCTC-3′.

Reverse: PfCpn20B: 5′-TTA CTCGAG ATATTTGGCCATGAGATA-3′.

The cleavage site of the signal sequence was chosen based on alignment with the sequence of the N-terminus of pea cpn20 [15]. The final construct contained the sequence of full-length mature Pf-cpn20 with a 6-histidine tag at the C-terminus and an additional leucine and glutamate at the end of the gene but before the histidines. The plasmid was transformed into *E. coli* BL21 tuner (Novagen) for expression of the Pf-cpn20 protein.

### Proteins

Bacterial growth, protein expression and purification of GroEL, GroES and At-cpn20 (At5g20720) were carried out as previously described [23]. Pf-cpn20 was purified based on the At-cpn20 purification protocol with an additional purification step, using a Superdex 200 gel filtration column (GE Healthcare) in Tris-HCl 20 mM pH 7.4, NaCl 200 mM and 5% glycerol. At-cpn10 (At2g44650) was purified as previously described [24].

All molar protein concentrations appearing in the paper refer to protomer concentrations.

### Analytical Ultracentrifugation

All experiments were carried out using an XL-I analytical ultracentrifuge (Beckman-Coulter Inc.), with a UV-visible optics detection system, using an An60Ti rotor and 12-mm double sector centerpieces. Sedimentation velocity analysis of the protein was carried out at 35,000 rpm at 20°C. The protein was equilibrated in 20 mM Tris-HCl pH 7.4 and 200 mM NaCl. The sedimentation velocity was carried out at a protein concentration of 0.5 mg/ml. Sedimentation profiles were registered every minute at 280 nm. The sedimentation coefficient distributions were calculated using the SEDFIT program [25], [26]. Sedimentation equilibrium analyses were carried out in buffer containing 20 mM Tris-HCl pH 7.4 and 200 mM NaCl, at 12,000 rpm at 20°C after equilibration for 10, 12 and 14 hrs. The equilibrium profile was registered at 280 nm. The molecular mass was calculated by the segal2.1 program [27]. The calculated molecular masses represent averages of three individual experiments with readings taken at three different time points each (10, 12 and 14 hours). For GroES, the equilibrium analysis was carried out once at 15,000 rpm in order to ensure that the system was working properly.

### Circular Dichroism

Measurements were carried out in a Chirascan™ CD (Circular Dichroism) spectrometer on 1 mg/ml protein in 5 mM NaHEPES pH 7.4 and 50 mM NaCl at a wavelength range of 190 nm –260 nm at a rate of 1 nm/2.5 sec, with a 0.01 cm path length. Five repeats of raw data were averaged, corrected by subtracting the contribution of the buffer to the CD signal, smoothed and converted to molar ellipticity. Possible effects of concentration on the CD profile were determined by measuring the CD spectrum of solutions containing 1, 1.5, 2 and 3 mg/ml Pf-cpn20 under the above conditions, in duplicate.

Temperature-dependent denaturation was carried out at 222 nm on solutions containing 0.3 mg/ml Pf-cpn20 or At-cpn20 in a 0.1 cm path-length cell. The temperature was increased from 5 to 79°C at 2°C intervals with a 180 sec setting time and 120 sec per point. T_m_ denotes the temperature at which the CD signal decreases to 50% of the maximum value at 222 nm.

### Light Scattering

Heat-induced aggregation of the proteins was confirmed through light scattering, by measuring the OD_600_ of Pf-cpn20 and At-cpn20 in a Cary 60 UV-Vis spectrophotometer (Agilent) at increasing temperatures. A 3 ml cuvette with a stirrer, containing 0.3 mg/ml Pf-cpn20 or At-cpn20 in buffer containing 5 mM NaHEPES pH 7.4 and 50 mM NaCl, was exposed to temperatures ranging from 25 to 80°C. Five minutes were allowed for temperature equilibration at each point and OD_600_ measurements were taken every 5°C.

### Protein Refolding Assays

Refolding of urea-denatured MDH was carried out as previously described [28]. Briefly, 35 µM malate dehydrogenase (MDH - Roche) was denatured in 4.5 M urea and 5 mM DTT for 3 hours at room temperature. The refolding reaction was carried out in two steps. First, MDH was rapidly diluted to 0.5 µM into a refolding solution containing 10 µM GroEL in 50 mM Tris-HCl (pH = 7.4), 10 mM MgCl_2_, 50 mM KCl, 5 mM DTT and incubated for 15 min at 30°C to form the chaperonin-protein binary complex. Next, the refolding of MDH was initiated by adding co-chaperonin at the indicated concentration and 5 mM ATP. Aliquots of 10 µl were removed at various times and assayed for MDH activity by adding 990 µl of reaction mixture containing 150 mM K-phosphate (pH = 7.5), 10 mM DTT, 0.28 mM NADH and 0.5 mM oxaloacetate and monitoring the oxidation of NADH at 340 nm. Refolding yields are expressed relative to the highest yield obtained using GroES.

### Cloning of Chaperonin-co-chaperonin Pairs into a pOFX Plasmid

Using the IPTG-inducible pOFX plasmid expressing wild-type human chaperonin and co-chaperonin [29] (mHsp60 and mHsp10), we engineered additional constructs containing various combinations of co-chaperonin and chaperonin from human, *E. coli*, *A. thaliana* and *P. falciparum* chaperonin systems. GroEL was cloned between the AflII and SpeI sites of pOFX using primers 1 & 2 (Table S1). Cloning of GroES, Pf-cpn20, At-cpn20 into pOFX was carried out in two steps, due to cloning constraints. First, a standard PCR reaction was carried out on either the ORF of GroES [30], or the DNA sequence of the predicted mature cpn20 genes, with primers 3&4, 5&6 or 7&8 for GroES, At-cpn20 or Pf-cpn20, respectively (Table S1). The PCR products were then digested with Eco105I and Eco81I and ligated into pOFX. This step resulted in co-chaperonin with an extension of three amino acids at its C-terminus. This extension was removed using site-directed PCR with primers 9&10, 11&12 or 13&14 for GroES, At-cpn20 or Pf-cpn20, respectively (Table S1).

### *In Vivo* Expression System

A system for investigating the ability of plasmid-borne chaperonin-co-chaperonin pairs to replace endogenous *E. coli* GroEL and GroES was previously described [30]–[33]. Briefly, the MGM100 strain of *E.coli* was used, in which the endogenous, GroEL-GroES chaperonin system was placed under strict control of the pBAD promoter [31], [32] requiring arabinose for induction. In our system, these bacteria were transformed with a pOFX plasmid which contained combinations of various cpn10 and cpn60 homologs under control of the lac promoter [29], [30] (see previous section). Upon transfer to medium containing only glucose and IPTG, but no arabinose, endogenous GroEL and GroES were not expressed, and only functional cpn10-cpn60 pairs on the pOFX plasmid were able to rescue the bacteria. As a negative control, a pOFX plasmid containing the mitochondrial chaperonin mHsp60 and GroES was used. Although the mHsp60-mHsp10 pair is able to replace the function of GroEL-GroES *in vivo* in *E. coli* [31], mHsp60 does not interact with GroES [34] and therefore, *E. coli* containing only mHsp60-GroES is not viable [30].

### Pull-down Experiments

30 µM of his-tagged Cpn20 (either At-cpn20 or Pf-cpn20), together with 20 µM of At-Cpn10, was incubated for 1 hour in 200 µl binding buffer composed of 50 mM Na-HEPES pH 7.7, 150 mM NaCl, 10 mM MgCl_2_, 100 mM KCl and 25 mM imidazole. After 1 hour incubation with 40 µl Ni-NTA beads (GE Healthcare) on an end-to-end shaker at room temperature, samples were centrifuged and washed 5 times with 200 µl binding buffer. The pellets, containing Ni-beads and associated proteins, were then suspended in 200 µl sample buffer and boiled for 10 min. Equivalent aliquots of 12 µl from the total sample, the unbound fraction, fourth wash and bound (pellet) fraction were analyzed by SDS-PAGE and stained with Coomassie Brilliant Blue R-250.

## Results

### Structural Properties of Pf-cpn20

As a baseline for our studies on the malarial co-chaperonin, we used the double co-chaperonin from *A. thaliana*, At-cpn20 which was shown to be similar in structure and function to the bacterial GroES [23]. An alignment of the two cpn20 sequences shows them to exhibit 24% identity and 57% similarity (based on identical and conserved amino acids in the mature protein) (Figure S1). It is interesting to note that the known structural and functional regions are not highly conserved at the level of the primary sequence (Figure S1) [10], [18]. In terms of secondary structure, cpn10 co-chaperonins were shown to exist as β-barrel structures, with the majority of the protein configured as anti-parallel β-sheets and random coils [35]–[38]. Consistent with what is known for cpn10 homologs, secondary structure prediction programs for the Pf-cpn20 sequence calculate a primarily β-sheet conformation (Figure S2) [10].

**Figure 1:**
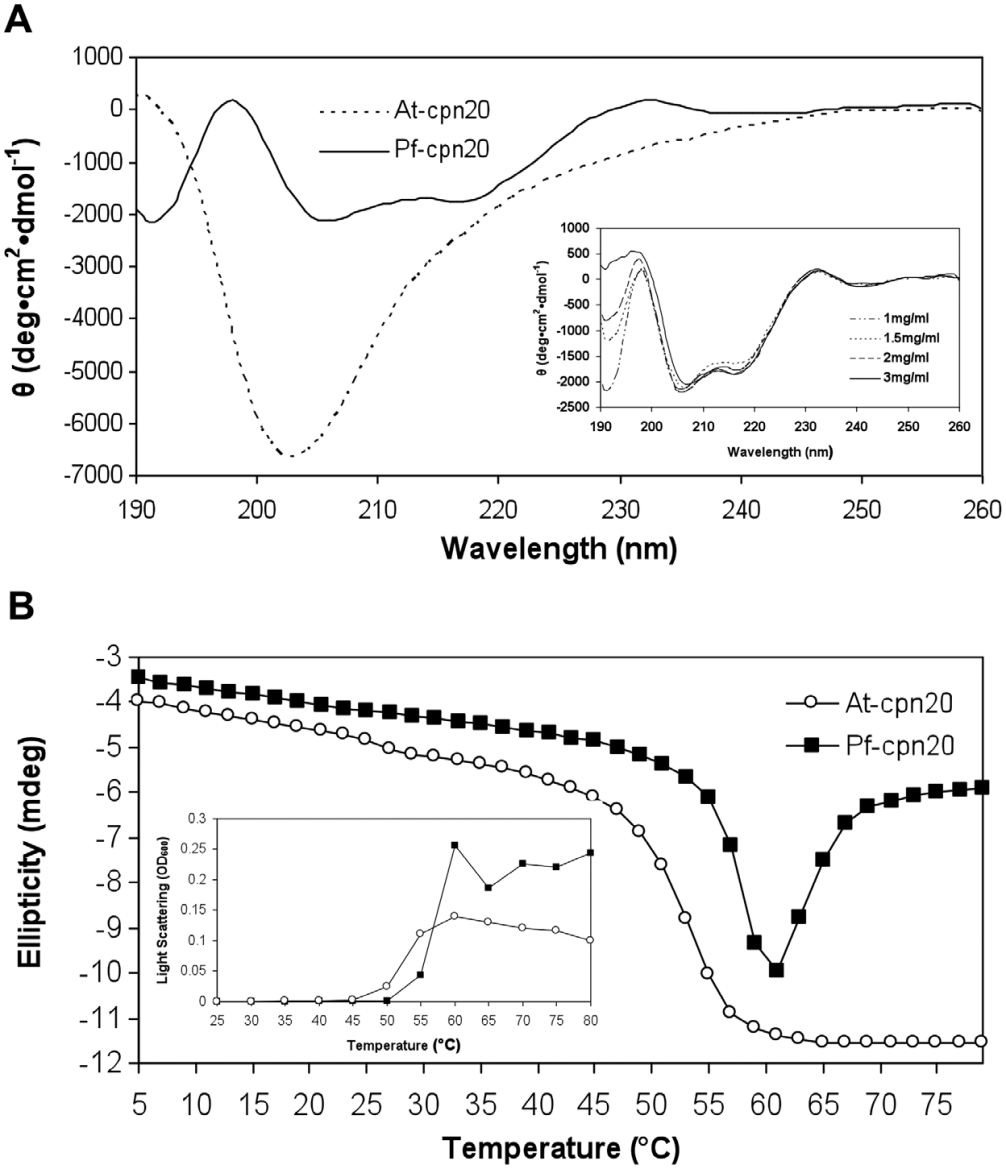
A circular dichroism analysis of the Pf-cpn20 structure in solution. Secondary structure characteristics of Pf-cpn20 were analyzed using a Chirascan™ spectrometer as described in Materials and Methods. A) The represented spectra of Pf- and At-cpn20 were obtained by averaging 5 spectra, correcting for buffer contribution and smoothing. Molar ellipticity as a function of wavelength is presented for each protein. Inset: The CD spectrum of solutions containing 1, 1.5, 2 and 3 mg/ml Pf-cpn20. B) Thermal denaturation curves were carried out for Pf- and At-cpn20 by varying the temperature from 5 to 79°C at 222 nm with 2°C intervals, 180 sec setting time and 120 sec time per point. Inset: Light scattering of Pf- and At-cpn20 measured at OD_600_ at increasing temperatures between 25 and 80°C.

Despite the large percentage of β-sheet, the salient CD signal of the bacterial co-chaperonin GroES in solution is consistent with that of an unordered polypeptide, with a negative extreme at approximately 200 nm [39]–[41]. This pattern was similar for other co-chaperonin homologs investigated in aqueous solution, including yeast cpn10 [42], *M. tuberculosis* cpn10 [43] and *A. thaliana* cpn20 [23]. In order to examine the secondary structure of *P. falciparum* cpn20 (Pf-cpn20) experimentally, we carried out CD measurements on recombinant Pf-cpn20, under conditions similar to those we used previously for analyzing the *A. thaliana* cpn20 (At-cpn20) [23]. A cursory glance at our data was enough to discern a very different CD spectrum for Pf-cpn20 than for At-cpn20 (Figure 1A). Additionally, Pf-cpn20 exhibited a significantly weaker CD signal. Even when the concentration was increased two and three fold in order to obtain a stronger signal, an identical pattern was observed, demonstrating that the overall structure is stable within this range (Figure 1A, inset). The CD spectrum for Pf-cpn20 exhibited a maximum at 198 nm and two minima, at 206 nm and 216 nm, compared to only one minimum for At-cpn20, at 204 nm. Despite these differences, the presence of a maximum at 195 nm and a minimum at 218 nm is consistent with a well-defined antiparallel β-pleated sheet [44]. Moreover, deconvolution of the data by the CDNN program [45], [46] revealed a secondary structure pattern that was almost identical between the two cpn20 homologs, and consistent with what would be expected for a typical cpn10 homolog of known β-barrel structure (Table 1) [35]–[38].

**Table 1:** Deconvolution of CD data for At-cpn20 and Pf-cpn20*.

Structure	At-cpn20	Pf-cpn20
Helix	8.90%	6.90%
Antiparallel	34.20%	37.70%
Parallel	3.50%	3.40%
Beta-Turn	23.10%	19.00%
Rndm. Coil	32.20%	33.10%
Total Sum	101.80%	100.20%

* Carried out on 1 mg/ml protein, 190–260 nm using the CDNN program [42], [43].

Next, we examined the thermal stability of Pf-cpn20 by following its CD signal at 222 nm at different temperatures. It can be seen in Figure 1B that Pf-cpn20 is relatively stable at high temperatures with a T_m_ of 55°C, while At-cpn20 exhibits a T_m_ of 51°C [23] and GroES is stable up to ∼70°C [47]. At high temperatures, the CD signal of At-cpn20 reached a minimum at approximately 60°C and remained constant up to the highest temperature examined (79°C). The CD signal of Pf-cpn20 reached a minimum at approximately 62°C and increased again upon a further increase in temperature. This change indicates an additional conformational change of the Pf-cpn20 at higher temperatures, possible reflecting aggregation of the denatured molecules [23], [28], [48]. In order to examine this possibility, we measured the light scattering of the two proteins at increasing temperatures. It can be seen that Pf-cpn20 and At-cpn20 both tend to aggregate at higher temperatures; however, the degree of aggregation is much greater for Pf-cpn20 than for At-cpn20 (Figure 1B, inset). This was confirmed by observation of the cuvette at the end of the experiment, in which aggregates of both proteins can be clearly seen. The aggregation was much more intense, however, in the cuvette containing Pf-cpn20 (Figure S3).

We carried out an analytical ultracentrifugation (AU) analysis, with the aim of characterizing the oligomeric species of Pf-cpn20 in solution. It should be noted that previous studies on the oligomeric structure of At-cpn20 showed it to be tetrameric based on experiments using chemical crosslinking combined with mass spectrometry and gel filtration [19]. Moreover, mobility in native gel electrophoresis was similar to that of GroES, suggesting that both proteins are characterized by a single species of 70–80 kDa [23]. However, a recent study using native spray mass spectrometry analysis showed that At-cpn20 on its own exists as a mixture of trimeric and tetrameric forms [20].

We carried out sedimentation velocity measurements, which showed At-cpn20 to be comprised of a single population with a sedimentation coefficient of 4S compared to Pf-cpn20, which revealed at least two detectable populations: 90% of the protein had a sedimentation coefficient of 4.5S and the remaining population had a sedimentation coefficient of 7.5S (Table 2). By way of comparison, GroES was shown to have a sedimentation coefficient of approximately 4 (Table 2) [49]–[51]. Using sedimentation equilibrium data, we calculated a molecular mass for Pf-cpn20 of 94.6±4.1 kDa, which is roughly consistent with a predicted tetrameric form (98 kDa). In comparison, the molecular mass of At-cpn20 was calculated to be 98±8.9 kDa, also suggesting a tetramer (90 kDa) (Table 2). Thus, the major population of cpn20 from both *A. thaliana* and *P. falciparum* gave a molecular mass that was consistent with a population of primarily tetrameric oligomers.

**Table 2:** Analytical ultracentrifugation values for GroES, At-cpn20 and Pf-cpn20.

Protein	Molecular Mass	s value(S)
GroES	72.6	4*
At-cpn20	98±8.9	4
Pf-cpn20	94.6±4.1	4.5 (90%), 7.5 (10%)

* [46]–[48].

### The Function of Pf-cpn20 *In Vitro* and *In Vivo*

**Figure 2:**
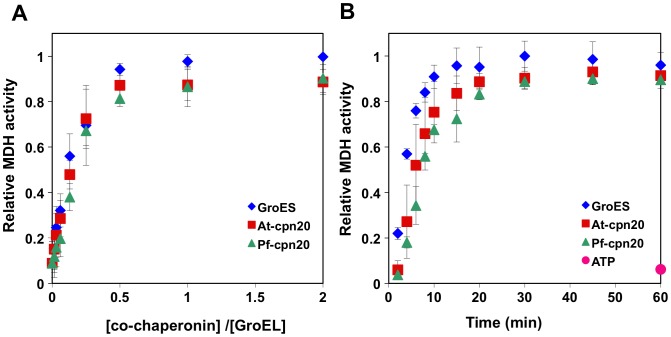
Refolding of denatured MDH by GroEL and Pf-cpn20. Urea-denatured malate dehydrogenase was refolded by GroEL with the help of Pf-cpn20, using At-cpn20 and GroES as control co-chaperonins. A) Refolding yields as a function of the co-chaperonin/GroEL protomer ratio. The refolding reaction was carried out for 30 minutes with 10 µM GroEL and increasing co-chaperonin concentration (0.16 to 20 µM), as described in Materials and Methods. Refolding is expressed relative to the highest yield obtained with GroES. B) Refolding yield as a function of time carried out with 10 µM GroEL and a sub-saturating co-chaperonin concentration (2 µM) Values represent the average of 3 independent experiments +/− standard deviation.

Alignment and sequence analysis of apicomplexan co-chaperonins revealed that the known structural features of co-chaperonins, specifically the mobile loop, roof domain and β-sheet structure, are retained in both GroES-like domains of the cpn20 [10]. Despite these similarities, it was reported that the Pf-cpn20 was unable to rescue two different *E coli* GroES mutant strains [10]. We set out to reexamine the protein-folding activity of Pf-cpn20 using two different approaches, *in vitro* with GroEL and *in vivo* in *E. coli*. In the first approach, we tested the ability of the purified Pf-cpn20 to act as a co-chaperonin, assisting GroEL at refolding MDH *in vitro*, using At-cpn20 and GroES as controls. This system is widely used as a model to demonstrate chaperonin function and GroEL was previously shown to be fully active with At-cpn20 [23]. As shown in Figure 2, the malarial co-chaperonin is fully functional and efficient at assisting GroEL in refolding of urea denatured MDH, when compared with GroES or At-cpn20. The protomer ratio of co-chaperonin to GroEL required for half maximal refolding yields was similar for all three systems (∼0.125 co-chaperonin to GroEL ratio) with maximal yields obtained at a 0.5 protomer ratio (Figure 2A). When refolding kinetics were measured at a suboptimal (0.2) ratio of co-chaperonin to chaperonin (2 µM/10 µM), a slight difference was observed between the three cpn10 homologs (Figure 2B). GroES exhibited the most rapid kinetics, followed by At-cpn20 and Pf-cpn20 with T_50_’s of 3.5, 5.5, and 7.5, minutes, respectively. A similar pattern is obtained using saturating amounts (20 µM) of co-chaperonin, although the difference in kinetics is less pronounced (Figure S4).

**Figure 3:**
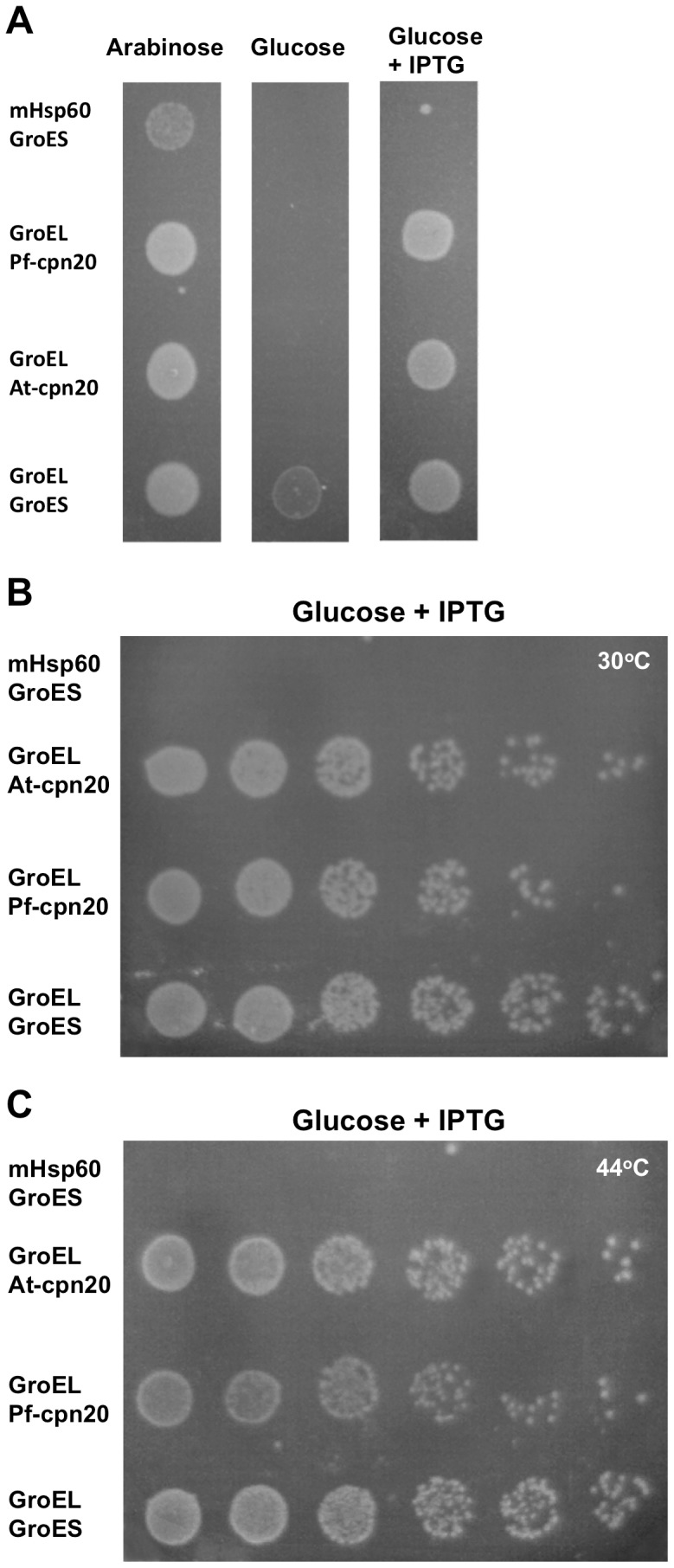
Pf-cpn20 successfully replaces the function of GroES in *E. coli*. Complementation assays were carried out in a strain of *E. coli*, MGM100, in which expression of endogenous chaperonins (GroEL-GroES) was under strict control of the pBAD promoter. (A) Various controls for the *in vivo* system at the indicated growth conditions (10^−2^ dilution shown). (B and C) Ten-fold-serial dilutions (10^−2^ to 10^−7^ shown) of *E. coli* strain MGM100 harboring plasmid pOFX containing GroEL and the indicated co-chaperonin, grown on agar plates in the presence of glucose and IPTG, but no arabinose: B) at 30°C C) at 44°C.

In the second approach, we tested the ability of Pf-cpn20 to complement a GroES depletion *in vivo* in an *E. coli* strain, in which endogenous chaperonin (GroEL-GroES) expression was under strict control of the pBAD promoter [31], [32]. Pf-Cpn20 was introduced together with GroEL on a pOFX plasmid under the control of an IPTG-inducible promoter and the *E. coli* strain was depleted of endogenous GroEL and GroES by growth in the absence of arabinose. Parallel control systems were set up in which either At-cpn20 or GroES was cloned into the pOFX plasmid together with GroEL. As a negative control, we used the mitochondrial chaperonin mHsp60 together with GroES, which do not interact and are unable to refold denatured protein or able to support growth of *E. coli* [30], [34]. All bacteria were able to grow in the presence of arabinose, but only those containing functional cpn60-cpn10 pairs were able to grow in the presence of IPTG (Figure 3A). Surprisingly, both At-cpn20 and Pf-cpn20 were fully able to rescue the bacteria together with GroEL. A small degree of growth was also observed in the absence of IPTG, in bacteria containing pOFX-GroEL-GroES, apparently due to leakiness of the plasmid, however, this was not enough to sustain bacteria containing plasmids coding for heterologous chaperonins (Figure 3A). Interestingly, we found that Pf-cpn20, as well as At-cpn20, was efficient at replacing the function of GroES in *E. coli*, at both 30°C (Figure 3B) and 44°C (Figure 3C), suggesting that the *P. falciparum* cpn20 is able to assist the folding of all GroEL substrates essential for growth, even under heat shock conditions. We conclude that *P. falciparum* cpn20 is a bona fide co-chaperonin.

### Can Pf-cpn20 Form Mixed Co-chaperonin Oligomers with Arabidopsis cpn10?

**Figure 4:**
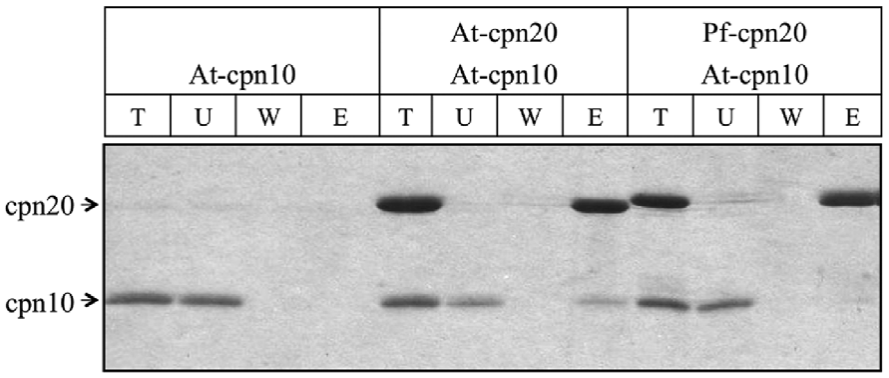
Pf-cpn20 does not form hetero-oligomers with At-cpn10. Interaction between Cpn20 and Cpn10 was measured using a pulldown assay. His-tagged At- or Pf-cpn20 was incubated with At-cpn10 and bound to Ni^2+^ beads as described in Materials and Methods. Equivalent aliquots of 12 µl from the total sample (T), unbound fraction (U), fourth wash (W), and bound fraction (B) were analyzed by SDS-PAGE and stained with Coomassie Brilliant Blue R-250.

A recent investigation revealed that cpn20 and cpn10 co-chaperonins from the single-celled algae *C. reinhardtii* are individually non-functional; only hetero-oligomers composed of specific combinations of the algal co-chaperonins cpn10, cpn20 and cpn23 are able to serve as co-chaperonins for GroEL [20]. The authors demonstrated that in *Arabidopsis* as well, three cpn20 subunits can form a functional hetero-oligomer with one cpn10 subunit. Based on the functional and structural similarity between Pf- and At-cpn20, it was interesting to determine whether the malarial co-chaperonin retains the ability to interact with cpn10. To this end, we carried out pull-down experiments using his-tagged Pf- and At-cpn20. As seen in Figure 4, Pf-cpn20 was much less able to pull down At-cpn10 than At-cpn20, if at all. These results suggest that, despite the great level of similarity at both the structural and functional levels, the lack of a cpn10 homolog in the apicoplast has resulted in reduction in the ability of Pf-cpn20 to bind cpn10.

## Discussion

Chloroplasts contain two types of co-chaperonins. One type is a classic, GroES-like co-chaperonin composed of 10 kDa subunits, known as cpn10. The second type, cpn20, is composed of two cpn10-like sequences fused head-to-tail and is found only in plant chloroplasts and in algae. Both cpn10 and cpn20 from *A. thaliana* were shown to form homo-oligomers that function as co-chaperonins in cpn60-mediated protein folding [15], [24], although a recent study showed that cpn20 and cpn10 in some species may also form active hetero-oligomers [20]. Since the malarial apicoplast contains only one co-chaperonin, cpn20 [9], [10], it must necessarily function as a homo-oligomer of 20 kDa subunits. However, the ability of this protein to assist in protein folding has not yet been demonstrated [10].

In this study, we revisited the function of apicoplast co-chaperonin using a two-fold approach. In the first, we expressed recombinant Pf-cpn20 in bacteria, purified it and examined its structural and functional properties *in vitro.* Our results indicate that the general structural properties of Pf-cpn20 (secondary structure, T_m_ and oligomeric state) are similar to its homolog found in *A. thaliana* chloroplasts. The only major structural differences that we observed were a higher propensity of Pf-cpn20 to aggregate at high temperatures, and the presence of a small amount of an additional oligomeric population. Still, the protein is thermally very stable, since its T_m_ value was 55°C, which is much higher than the temperatures that the parasite will encounter during its life cycle. In terms of *in vitro* function, the malarial cpn20 protein was fully able to function with GroEL at refolding denatured MDH, with only a slight difference in refolding kinetics seen at sub-optimal co-chaperonin/chaperonin ratios.

Stoichiometric analyses of this system present a challenge, since the actual functional form of these co-chaperonins remains a mystery. All known type I cpn60 chaperonins can be described by heptameric symmetry and were shown to interact with heptameric co-chaperonin rings. However, At-cpn20 was originally reported to be a tetramer [16], [19] and was demonstrated to be functional in this form [23]. The difficulty in reconciling 7 co-chaperonin binding sites of a cpn60 ring with the 8 potentially-interacting mobile loops of a tetrameric cpn20 was tentatively solved by Tsai et al. [20]. These authors showed that cpn20 and cpn10 monomers could combine to form functional hetero-oligomers with 7 cpn10 domains, in both *C. reinhardtii* and *A. thaliana*. Functional, homo-oligomeric At-cpn20 was shown to bind chaperonin by excluding the 8^th^ domain from interaction with cpn60. Since the *P. falciparum* apicoplast proteome is predicted to have only one co-chaperonin protein, no single-domained cpn10 exists with which to form heptameric hetero-oligomers, thus implying a mechanism of 8^th^–domain exclusion. Sense might be made of this scenario by considering an additional role for the excluded domain(s) of cpn20, as previously suggested [18]. Indeed, At-cpn20 was recently shown to mediate iron superoxide dismutase (FeSOD) activity in the *A. thaliana* chloroplast, independent of its co-chaperonin role [52].

Our *in vitro* refolding studies showed a very similar protomer-concentration-dependence for the activity of GroES and both cpn20’s. If indeed cpn20 functions as a tetramer, this would imply almost twice as much (7/4 = 1.75) cpn20 oligomer needed compared to GroES oligomer, in order to achieve a similar refolding yield and would indicate a lower affinity of the cpn20’s for GroEL. However, we still cannot rule out the possibility that some kind of heptameric form of cpn20 is interacting with the chaperonin.

In the second approach, we expressed Pf-cpn20 in *E. coli* as the sole co-chaperonin. We were surprised to find that Pf-cpn20 was fully functional as a co-chaperonin and successfully replaced the function of GroES *in vivo*. The ability to functionally express malarial cpn20 in *E. coli* will provide an important tool for studying this protein, as well as its interactions with other proteins, or chemical compounds. Many antibiotics known today are used as anti-malarial treatments, and induce what is known as “delayed death”, in which parasites survive for one generation, but die out in the second generation [53]. Analysis of these compounds showed that they target the apicoplast. Although many of these compounds were shown to inhibit apicoplast biogenesis and function, none are known to function by inhibiting protein folding in the apicoplast [53], [54]. Since the double structure of cpn20 is specific to only eukaryotes of plant lineages and is preferably conserved throughout evolution over the smaller, single-domained, cpn10 protein, it may constitute a useful drug target for use in various types of malarial therapy. Our success in showing, for the first time, that Pf-cpn20 can function *in vitro* with GroEL and be expressed in *E. coli* as a functional co-chaperonin paves the way for this type of research.

## Supporting Information

Figure S1 **Sequence alignment of full-length cpn20 protein from** ***A. thaliana*** **and** ***P. falciparum*** **, carried out using ClustalW.** Orange signifies the putative transit peptides, green the linker region, blue the mobile loops and lavender the roof loops. Identical amino acids are marked with a star (*), conserved amino acids with a colon (**:**), and semi-conserved amino acids with a period (**.**).
(PPT)

Figure S2 **Secondary structure prediction of the Pf-cpn20 sequence using Psipred 3** **[55]**, **[56]** **.**
(PPT)

Figure S3 **Aggregation of cpn20 following exposure to high temperature.** Pf-cpn20 and At-cpn20 were exposed to increasing temperatures (25–80°C) as described. The cuvettes were photographed at the end of the experiment and are presented to allow visualization of the degree of aggregation.
(PPT)

Figure S4 **Time-dependent refolding of denatured MDH by GroEL and Pf-cpn20.** Urea-denatured malate dehydrogenase was refolded by GroEL (10 µM) with the help of 20 µM Pf-cpn20, At-cpn20 or GroES. The refolding reaction was carried out as described in Materials and Methods. Refolding yields are expressed as the activity obtained relative to the highest value obtained with GroES. Values represent the average of 4 independent experiments +/− standard deviation.
(PPT)

Table S1 **Primers used for cloning of constructs for in vivo analysis.**
(DOC)
