# Supplementary material for: P. falciparum cpn20 Is a Bona Fide Co-Chaperonin That Can Replace GroES in E. coli
Source: PLoS One. 2013 Jan 10;8(1):e53909. doi: 10.1371/journal.pone.0053909 (PMC3542282; doi:10.1371/journal.pone.0053909)
Supplement: Figure S1 — Sequence alignment of full-length cpn20 protein from A. thaliana and P. falciparum , carried out using ClustalW. Orange signifies the putative transit peptides, green the linker region, blue the mobile loops and lavender the roof loops. Identical amino acids are marked with a star (*), conserved amino acids with a colon (:), and semi-conserved amino acids with a period (.). (PPT) [file pone.0053909.s001.ppt]

## Slide 1
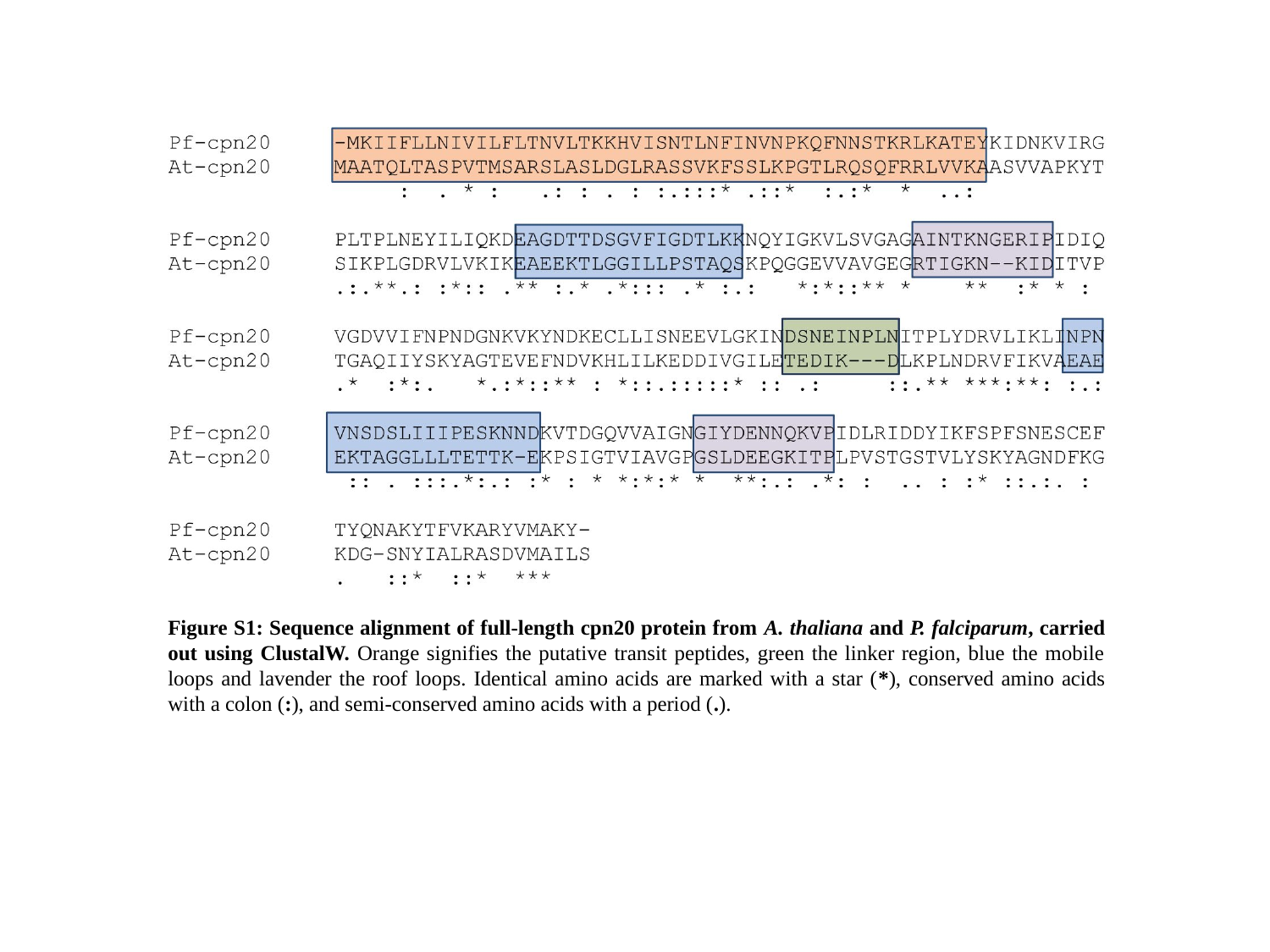

Figure S1: Sequence alignment of full-length cpn20 protein from A. thaliana and P. falciparum, carried out using ClustalW. Orange signifies the putative transit peptides, green the linker region, blue the mobile loops and lavender the roof loops. Identical amino acids are marked with a star (*), conserved amino acids with a colon (:), and semi-conserved amino acids with a period (.).
