# Supplementary figures and images for: P. falciparum cpn20 Is a Bona Fide Co-Chaperonin That Can Replace GroES in E. coli
Source: PLoS One. 2013 Jan 10;8(1):e53909. doi: 10.1371/journal.pone.0053909 (PMC3542282; doi:10.1371/journal.pone.0053909)

## Slide 1
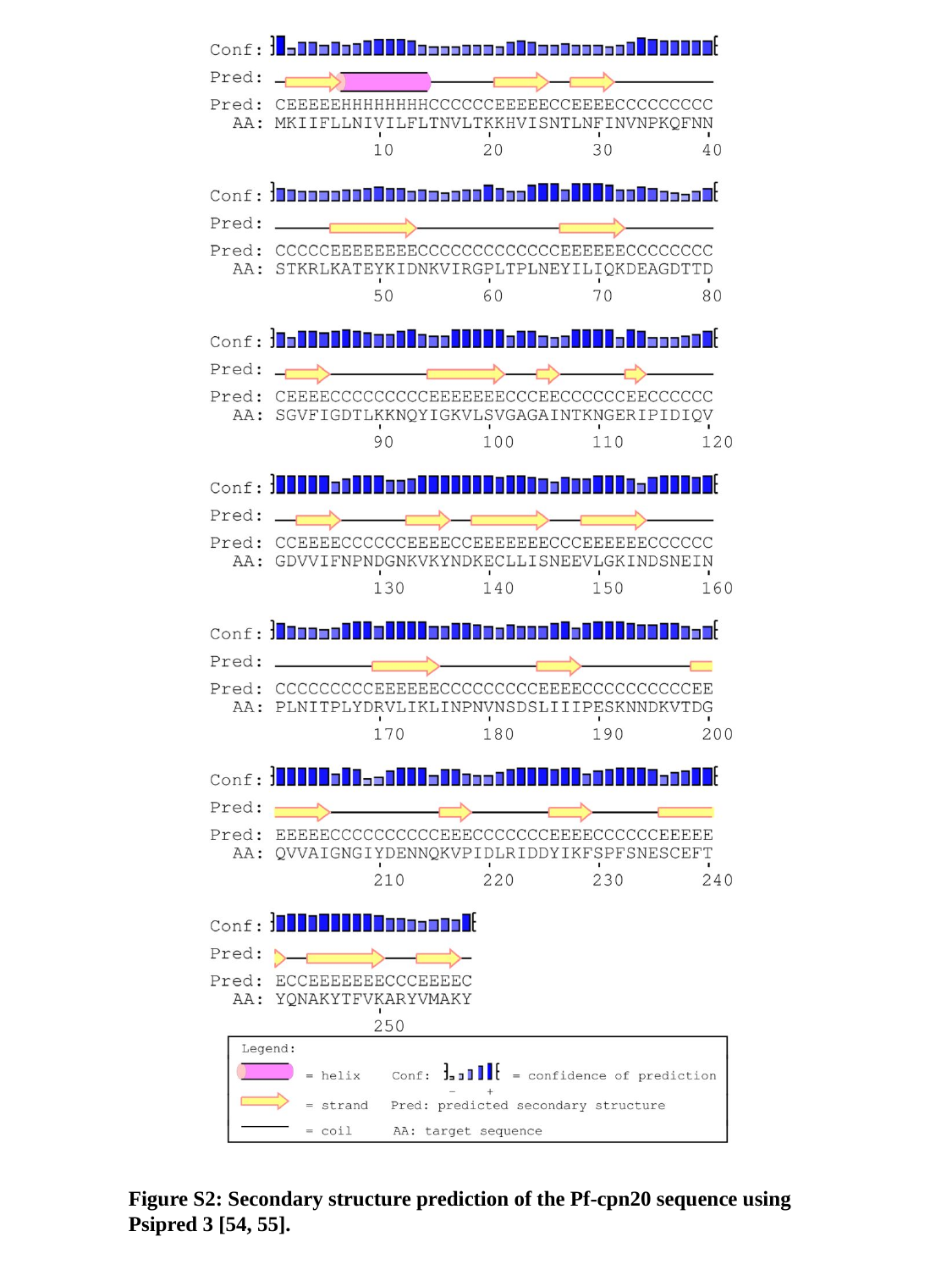

Figure S2: Secondary structure prediction of the Pf-cpn20 sequence using Psipred 3 [54, 55].

Supplement: Figure S2 — Secondary structure prediction of the Pf-cpn20 sequence using Psipred 3 [55] , [56] . (PPT) [file pone.0053909.s002.ppt]
