# Supplementary material for: P. falciparum cpn20 Is a Bona Fide Co-Chaperonin That Can Replace GroES in E. coli
Source: PLoS One. 2013 Jan 10;8(1):e53909. doi: 10.1371/journal.pone.0053909 (PMC3542282; doi:10.1371/journal.pone.0053909)
Supplement: Figure S3 — Aggregation of cpn20 following exposure to high temperature. Pf-cpn20 and At-cpn20 were exposed to increasing temperatures (25–80°C) as described. The cuvettes were photographed at the end of the experiment and are presented to allow visualization of the degree of aggregation. (PPT) [file pone.0053909.s003.ppt]

## Slide 1
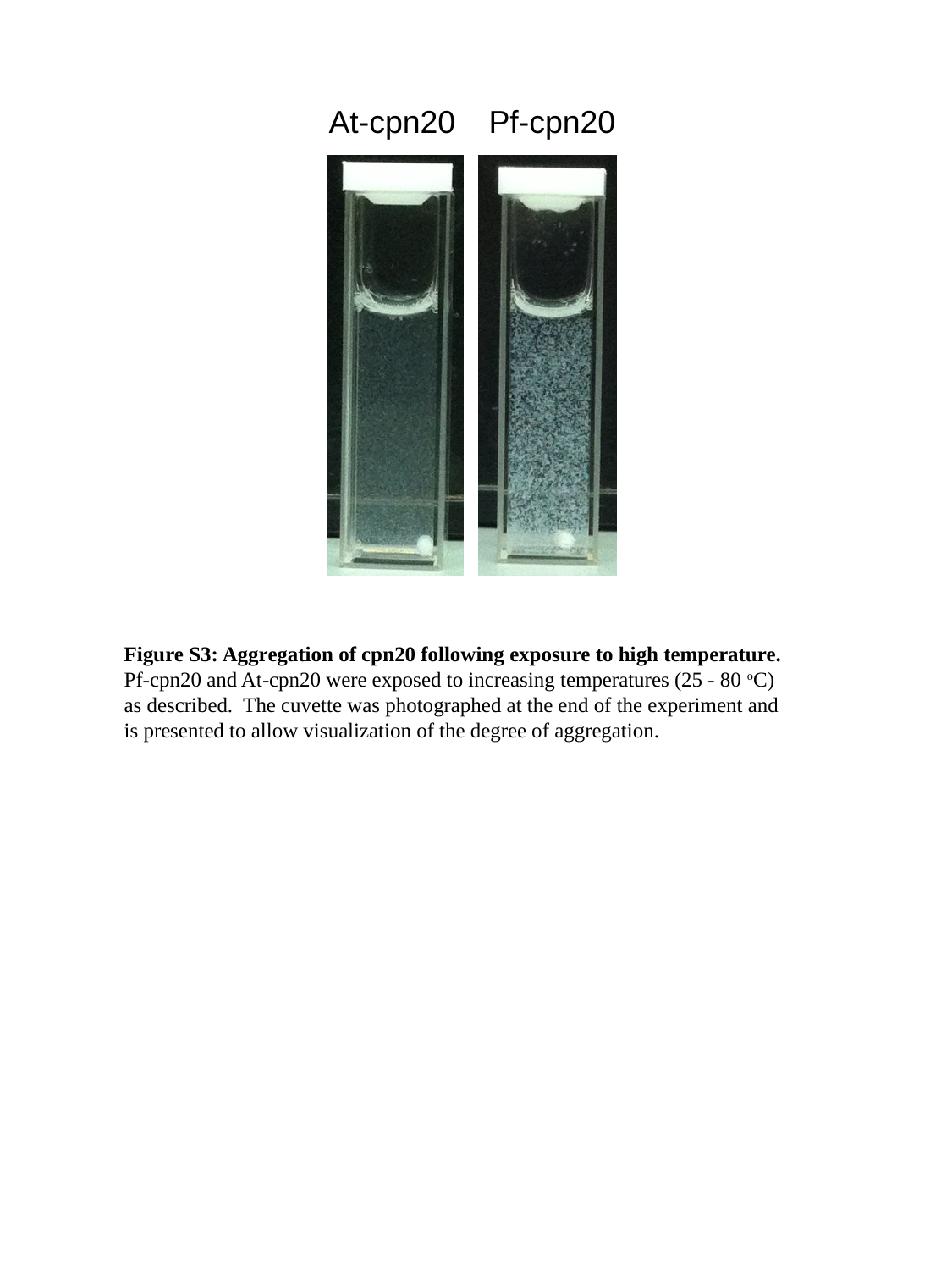

At-cpn20
Pf-cpn20
Figure S3: Aggregation of cpn20 following exposure to high temperature.
Pf-cpn20 and At-cpn20 were exposed to increasing temperatures (25 - 80 oC)
as described. The cuvette was photographed at the end of the experiment and
is presented to allow visualization of the degree of aggregation.
