# Supplementary material for: P. falciparum cpn20 Is a Bona Fide Co-Chaperonin That Can Replace GroES in E. coli
Source: PLoS One. 2013 Jan 10;8(1):e53909. doi: 10.1371/journal.pone.0053909 (PMC3542282; doi:10.1371/journal.pone.0053909)
Supplement: Figure S4 — Time-dependent refolding of denatured MDH by GroEL and Pf-cpn20. Urea-denatured malate dehydrogenase was refolded by GroEL (10 µM) with the help of 20 µM Pf-cpn20, At-cpn20 or GroES. The refolding reaction was carried out as described in Materials and Methods. Refolding yields are expressed as the activity obtained relative to the highest value obtained with GroES. Values represent the average of 4 independent experiments +/− standard deviation. (PPT) [file pone.0053909.s004.ppt]

## Slide 1
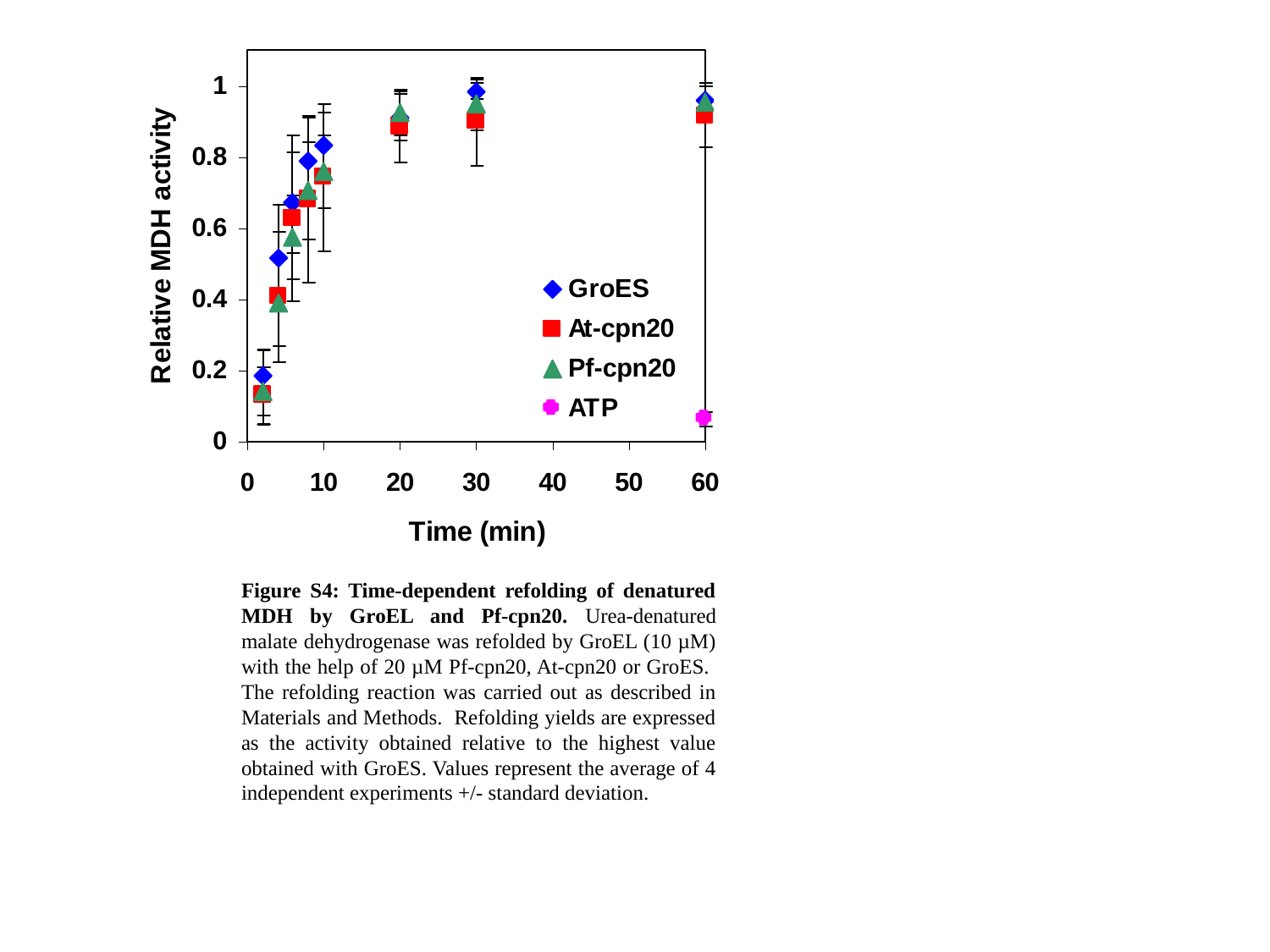

Figure S4: Time-dependent refolding of denatured MDH by GroEL and Pf-cpn20. Urea-denatured malate dehydrogenase was refolded by GroEL (10 µM) with the help of 20 µM Pf-cpn20, At-cpn20 or GroES. The refolding reaction was carried out as described in Materials and Methods. Refolding yields are expressed as the activity obtained relative to the highest value obtained with GroES. Values represent the average of 4 independent experiments +/- standard deviation.
