# Supplementary material for: P. falciparum cpn20 Is a Bona Fide Co-Chaperonin That Can Replace GroES in E. coli
Source: PLoS One. 2013 Jan 10;8(1):e53909. doi: 10.1371/journal.pone.0053909 (PMC3542282; doi:10.1371/journal.pone.0053909)
Supplement: Table S1 — Primers used for cloning of constructs for in vivo analysis. (DOC) [file pone.0053909.s005.doc]

**Table S1: Primers used for cloning of constructs for *in vivo* analysis**

|  | **Primer Name** | **Sequence** |
| --- | --- | --- |
| 1 | AflII_GroEL | AAT CTT AAG GAG GTC GGG CTA TGG CAG CTAA AGA C |
| 2 | GroEL_SpeI | TAT ACT AGT CTA GGA GTT ACA TCA TGC CGC CCA T |
| 3 | Eco81I_GroES | TAT CCT AAG GAG GTG GAG TAA TGA ATA TTC GTC CAT |
| 4 | GroES_Eco105I | TAA TAC GTA CGC TTC AAC AAT TGC CAG |
| 5 | CPN20- N-Eco81I | TAT CCT AAG GAG GTG GAG TAA TGG CTT CTG TTG TTG |
| 6 | CPN20- C-Eco105I | GCG TAC GTA AGA AAG TAT AGC CAT CAC |
| 7 | Pf-cpn20-N-Eco81I | TAT CCT AAG GAG GTG GAG TAA TGT ATA AAA TTG ATA ATA AAG |
| 8 | Pf-cpn20-C-Eco105I | GCG TAC GTA ATA TTT GGC CATG ACA TAT CTA |
| 9 | 9del_SENSE (GroES) | CTG GCA ATT GTT GAA GCG TGA AAT AAG TCA CTA TTG |
| 10 | 9del_ANTISENSE (GroES) | CAA TAG TGA CTT ATT TCA CGC TTC AAC AAT TGC CAG |
| 11 | 9del_cpn20_sense | GTG ATG GCT ATA CTT TCT TGA AAT AAG TCA C |
| 12 | 9del_cpn20_antisense | GTG ACT TAT TTC AAG AAA GTA TAG CCA TCA C |
| 13 | Pf-cpn20-del-F | GTC ATG GCC AAA TAT TGA AAT AAG TCA CTA TTG |
| 14 | Pf-cpn20-del-R | CAA TAG TGA CTT ATT TCA ATA TTT GGC CAT GAC |
